# Supplementary material for: Comparative transcriptomics of Venus flytrap (Dionaea muscipula) across stages of prey capture and digestion
Source: PLoS One. 2024 Aug 12;19(8):e0305117. doi: 10.1371/journal.pone.0305117 (PMC11318880; doi:10.1371/journal.pone.0305117)
Supplement: S1 Fig — Gene ontology dotplots including: (1) Top 30 Enriched Gene Ontology Biological Process Terms for differentially expressed genes between prey/no prey traps at 1 hr. The Rich Factor is the ratio of the number of enriched DEGs in the GO BP category. Point size shows the number of genes assigned to each GO category. The -log10(P value) is represented by a color scale. (2) Top 30 Enriched Gene Ontology Molecular Function Terms for differentially expressed genes between prey/no prey traps at 1 hr. The Rich Factor is the ratio of the number of enriched DEGs in the GO MF category. Point size shows the number of genes assigned to each GO category. The -log10(P value) is represented by a color scale. (3) Top 19 Enriched Gene Ontology Cellular Component for differentially expressed genes between prey/no prey traps at 1 hr. The Rich Factor is the ratio of the number of enriched DEGs in the GO CC category. Point size shows the number of genes assigned to each GO category. The -log10(P value) is represented by a color scale. (4) Top 30 Enriched Gene Ontology Biological Process Terms for differentially expressed genes between prey/no prey traps at 24 hr. The Rich Factor is the ratio of the number of enriched DEGs in the GO BP category. Point size shows the number of genes assigned to each GO category. The -log10(P value) is represented by a color scale. (5) Top 30 Enriched Gene Ontology Molecular Function Terms for differentially expressed genes between prey/no prey traps at 24 hr. The Rich Factor is the ratio of the number of enriched DEGs in the GO MF category. Point size shows the number of genes assigned to each GO category. The -log10(P value) is represented by a color scale. (6) Top 23 Enriched Gene Ontology Cellular Component for differentially expressed genes between prey/no prey traps at 1 hr. The Rich Factor is the ratio of the number of enriched DEGs in the GO CC category. Point size shows the number of genes assigned to each GO category. The -log10(P value) is represen [file pone.0305117.s001.pdf]

Supplemental Information 1. Gene ontology dotplots including: (1) Top 30 Enriched Gene Ontology Biological Process Terms for differentially expressed genes between prey/no prey traps at 1 hr. The Rich Factor is the ratio of the number of enriched DEGs in the GO BP category. Point size shows the number of genes assigned to each GO category. The -log10(P value) is represented by a color scale. (2) Top 30 Enriched Gene Ontology Molecular Function Terms for differentially expressed genes between prey/no prey traps at 1 hr. The Rich Factor is the ratio of the number of enriched DEGs in the GO MF category. Point size shows the number of genes assigned to each GO category. The -log10(P value) is represented by a color scale. (3) Top 19 Enriched Gene Ontology Cellular Component for differentially expressed genes between prey/no prey traps at 1 hr. The Rich Factor is the ratio of the number of enriched DEGs in the GO CC category. Point size shows the number of genes assigned to each GO category. The -log10(P value) is represented by a color scale. (4) Top 30 Enriched Gene Ontology Biological Process Terms for differentially expressed genes between prey/no prey traps at 24 hr. The Rich Factor is the ratio of the number of enriched DEGs in the GO BP category. Point size shows the number of genes assigned to each GO category. The -log10(P value) is represented by a color scale. (5) Top 30 Enriched Gene Ontology Molecular Function Terms for differentially expressed genes between prey/no prey traps at 24 hr. The Rich Factor is the ratio of the number of enriched DEGs in the GO MF category. Point size shows the number of genes assigned to each GO category. The -log10(P value) is represented by a color scale.(6) Top 23 Enriched Gene Ontology Cellular Component for differentially expressed genes between prey/no prey traps at 1 hr. The Rich Factor is the ratio of the number of enriched DEGs in the GO CC category. Point size shows the number of genes assigned to each GO category. The -log10(P value) is represented by a color scale.

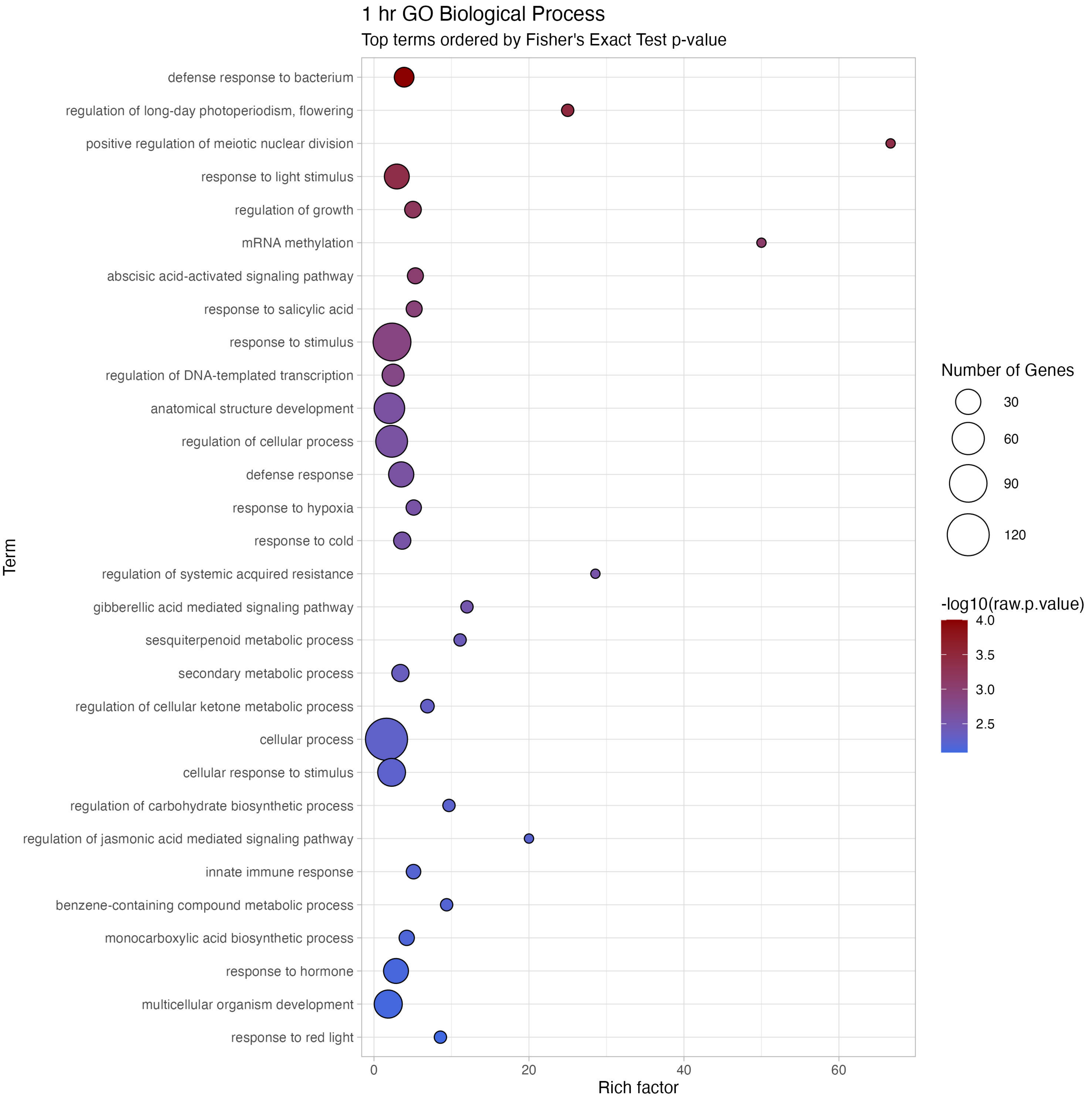

1 hr GO Biological Process
Top terms ordered by Fisher's Exact Test p-value
defense response to bacterium
regulation of long-day photoperiodism, flowering
positive regulation of meiotic nuclear division
response to light stimulus
regulation of growth
mRNA methylation
abscisic acid-activated signaling pathway
response to salicylic acid
response to stimulus
regulation of DNA-templated transcription
anatomical structure development
regulation of cellular process
defense response
response to hypoxia
response to cold
regulation of systemic acquired resistance
gibberellic acid mediated signaling pathway
sesquiterpenoid metabolic process
secondary metabolic process
regulation of cellular ketone metabolic process
cellular process
cellular response to stimulus
regulation of carbohydrate biosynthetic process
regulation of jasmonic acid mediated signaling pathway
innate immune response
benzene-containing compound metabolic process
monocarboxylic acid biosynthetic process
response to hormone
multicellular organism development
response to red light
Term
0
20
40
60
Rich factor
Number of Genes
30
60
90
120
-log10(raw.p.value)
4.0
3.5
3.0
2.5

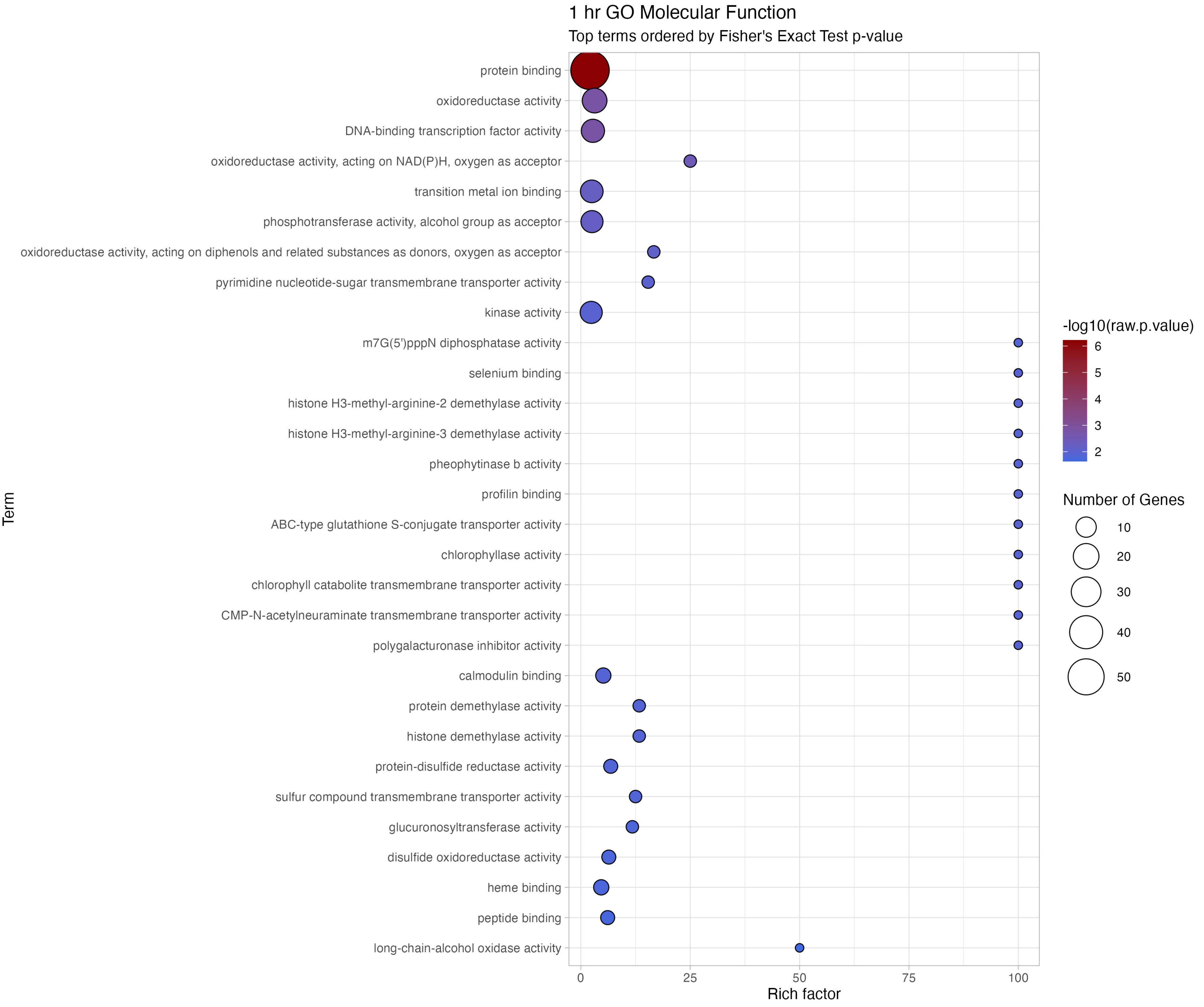
1 hr GO Molecular Function
Top terms ordered by Fisher's Exact Test p-value
protein binding
oxidoreductase activity
DNA-binding transcription factor activity
oxidoreductase activity, acting on NAD(P)H, oxygen as acceptor
transition metal ion binding
phosphotransferase activity, alcohol group as acceptor
oxidoreductase activity, acting on diphenols and related substances as donors, oxygen as acceptor
pyrimidine nucleotide-sugar transmembrane transporter activity
kinase activity
m7G(5')pppN diphosphatase activity
selenium binding
histone H3-methyl-arginine-2 demethylase activity
histone H3-methyl-arginine-3 demethylase activity
pheophytinase b activity
profilin binding
ABC-type glutathione S-conjugate transporter activity
chlorophyllase activity
chlorophyll catabolite transmembrane transporter activity
CMP-N-acetylneuraminate transmembrane transporter activity
polygalacturonase inhibitor activity
calmodulin binding
protein demethylase activity
histone demethylase activity
protein-disulfide reductase activity
sulfur compound transmembrane transporter activity
glucuronosyltransferase activity
disulfide oxidoreductase activity
heme binding
peptide binding
long-chain-alcohol oxidase activity
Term
0
25
50
75
100
Rich factor
-log10(raw.p.value)
6
5
4
3
2
Number of Genes
10
20
30
40
50

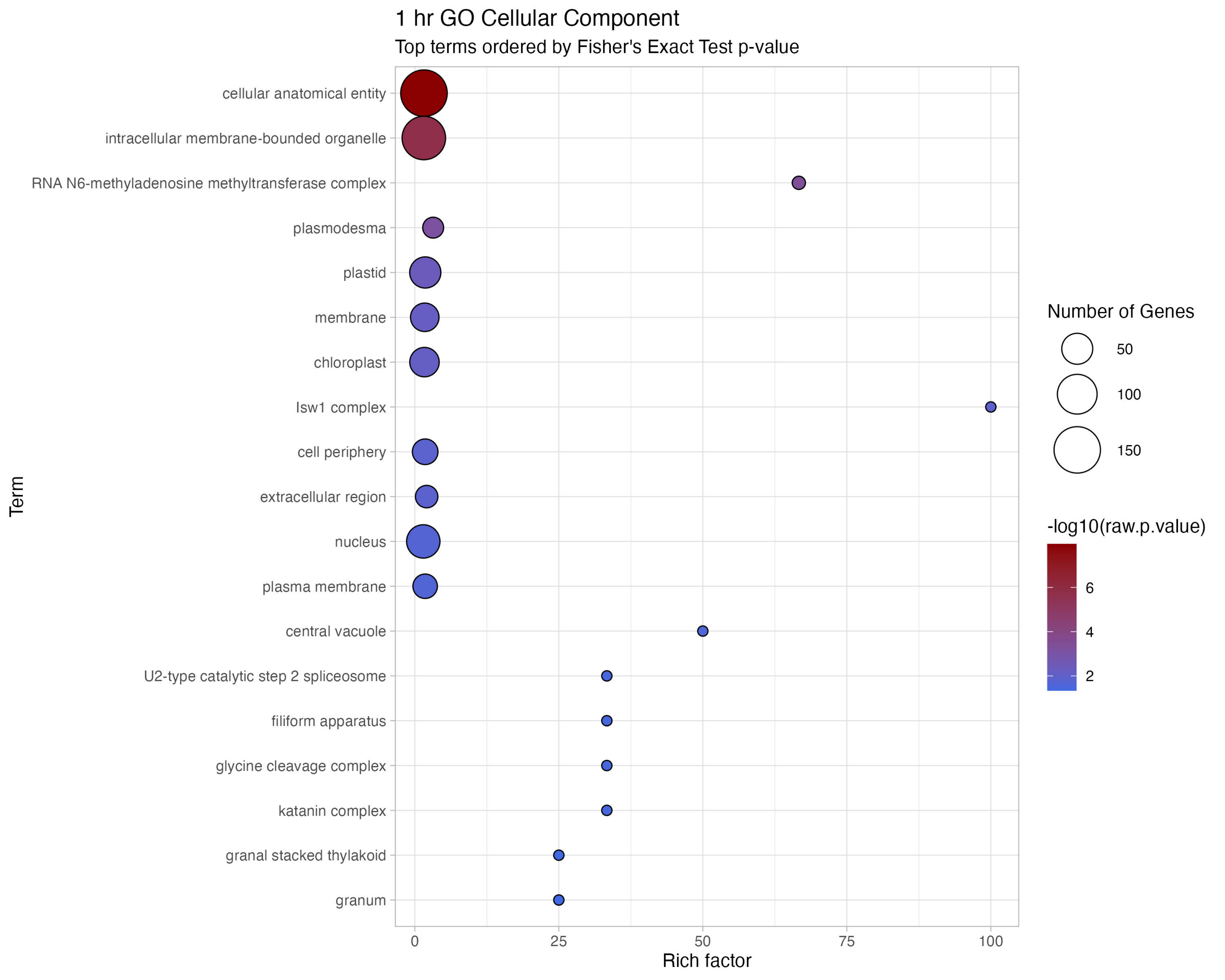
1 hr GO Cellular Component
Top terms ordered by Fisher's Exact Test p-value
cellular anatomical entity
intracellular membrane-bounded organelle
RNA N6-methyladenosine methyltransferase complex
plasmodesma
plastid
membrane
chloroplast
Isw1 complex
cell periphery
extracellular region
nucleus
plasma membrane
central vacuole
U2-type catalytic step 2 spliceosome
filiform apparatus
glycine cleavage complex
katanin complex
granal stacked thylakoid
granum
Term
0
25
50
75
100
Rich factor
Number of Genes
50
100
150
-log10(raw.p.value)
6
4
2

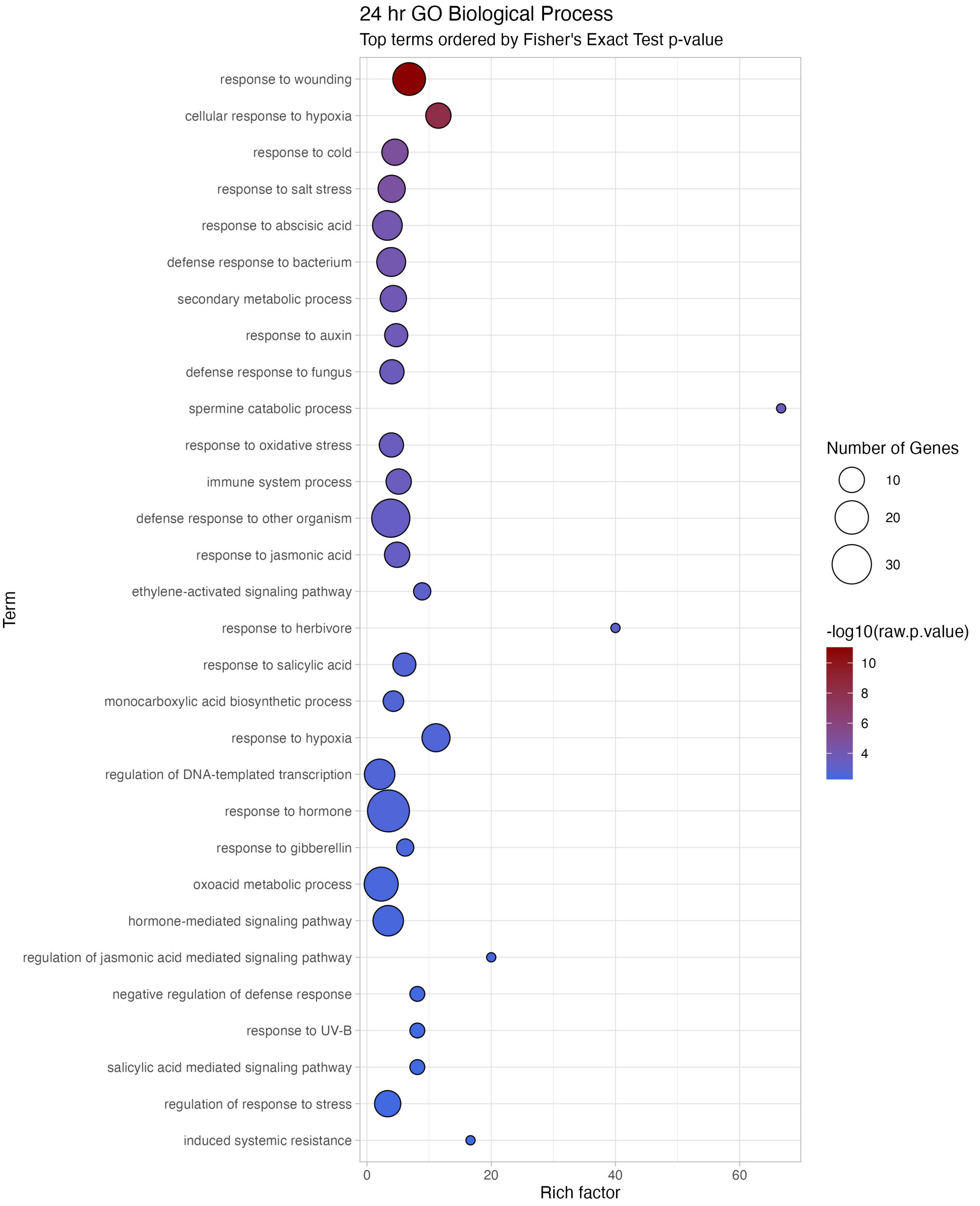

24 hr GO Biological Process
Top terms ordered by Fisher's Exact Test p-value
response to wounding
cellular response to hypoxia
response to cold
response to salt stress
response to abscisic acid
defense response to bacterium
secondary metabolic process
response to auxin
defense response to fungus
spermine catabolic process
response to oxidative stress
immune system process
defense response to other organism
response to jasmonic acid
ethylene-activated signaling pathway
response to herbivore
response to salicylic acid
monocarboxylic acid biosynthetic process
response to hypoxia
regulation of DNA-templated transcription
response to hormone
response to gibberellin
oxoacid metabolic process
hormone-mediated signaling pathway
regulation of jasmonic acid mediated signaling pathway
negative regulation of defense response
response to UV-B
salicylic acid mediated signaling pathway
regulation of response to stress
induced systemic resistance
Term
0
20
40
60
Rich factor
Number of Genes
10
20
30
-log10(raw.p.value)
10
8
6
4

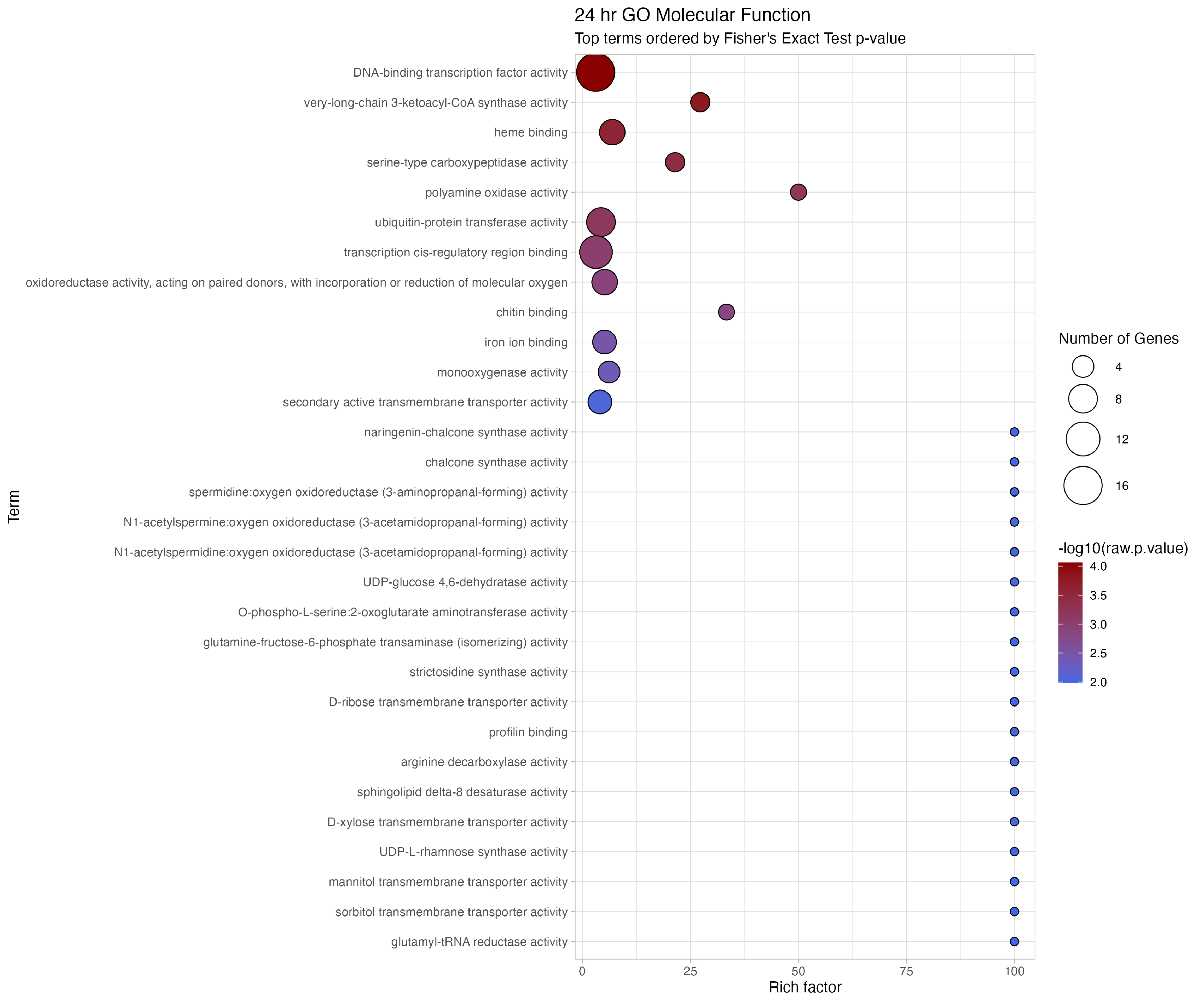
24 hr GO Molecular Function
Top terms ordered by Fisher's Exact Test p-value
DNA-binding transcription factor activity
very-long-chain 3-ketoacyl-CoA synthase activity
heme binding
serine-type carboxypeptidase activity
polyamine oxidase activity
ubiquitin-protein transferase activity
transcription cis-regulatory region binding
oxidoreductase activity, acting on paired donors, with incorporation or reduction of molecular oxygen
chitin binding
iron ion binding
monooxygenase activity
secondary active transmembrane transporter activity
naringenin-chalcone synthase activity
chalcone synthase activity
spermidine:oxygen oxidoreductase (3-aminopropanal-forming) activity
N1-acetylspermine:oxygen oxidoreductase (3-acetamidopropanal-forming) activity
N1-acetylspermidine:oxygen oxidoreductase (3-acetamidopropanal-forming) activity
UDP-glucose 4,6-dehydratase activity
O-phospho-L-serine:2-oxoglutarate aminotransferase activity
glutamine-fructose-6-phosphate transaminase (isomerizing) activity
strictosidine synthase activity
D-ribose transmembrane transporter activity
profilin binding
arginine decarboxylase activity
sphingolipid delta-8 desaturase activity
D-xylose transmembrane transporter activity
UDP-L-rhamnose synthase activity
mannitol transmembrane transporter activity
sorbitol transmembrane transporter activity
glutamyl-tRNA reductase activity
Term
0
25
50
75
100
Rich factor
Number of Genes
4
8
12
16
-log10(raw.p.value)
4.0
3.5
3.0
2.5
2.0

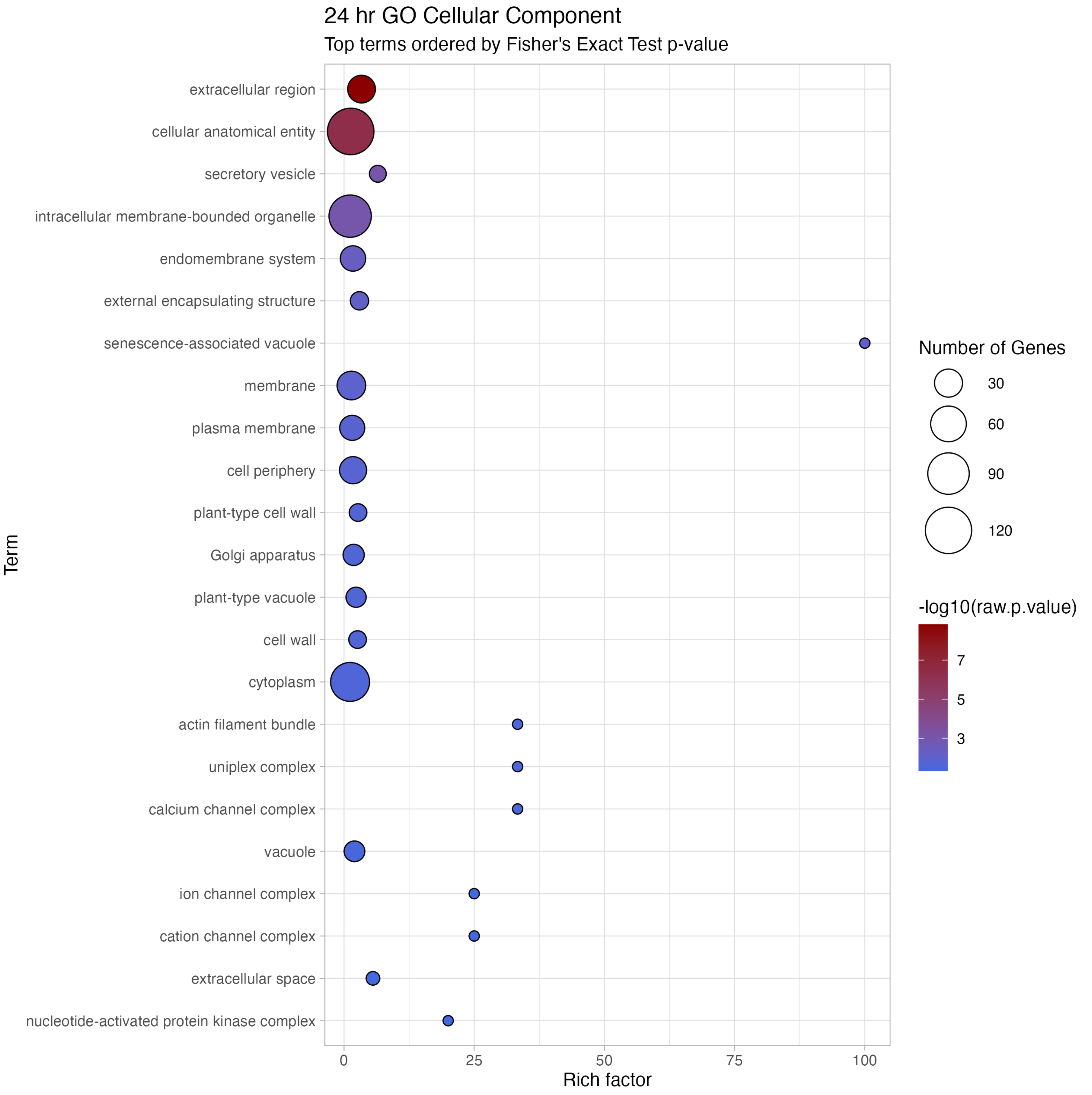
24 hr GO Cellular Component
Top terms ordered by Fisher's Exact Test p-value
extracellular region
cellular anatomical entity
secretory vesicle
intracellular membrane-bounded organelle
endomembrane system
external encapsulating structure
senescence-associated vacuole
membrane
plasma membrane
cell periphery
plant-type cell wall
Golgi apparatus
plant-type vacuole
cell wall
cytoplasm
actin filament bundle
uniplex complex
calcium channel complex
vacuole
ion channel complex
cation channel complex
extracellular space
nucleotide-activated protein kinase complex
Term
0
25
50
75
100
Rich factor
Number of Genes
30
60
90
120
-log10(raw.p.value)
7
5
3
