# Supplementary material for: Comparative transcriptomics of Venus flytrap (Dionaea muscipula) across stages of prey capture and digestion
Source: PLoS One. 2024 Aug 12;19(8):e0305117. doi: 10.1371/journal.pone.0305117 (PMC11318880; doi:10.1371/journal.pone.0305117)
Supplement: S2 Fig — Clusters represent co-expressed genes within each module by treatment (prey vs. no prey). Mean expression is shown in the black line. (PDF) [file pone.0305117.s002.pdf]

Supplemental Information 2. Expression Z-scores for each gene in each of the modules with >5 genes (n=14). Clusters represent co-expressed genes within each module by treatment (prey vs. no prey). Mean expression is shown in the black line.

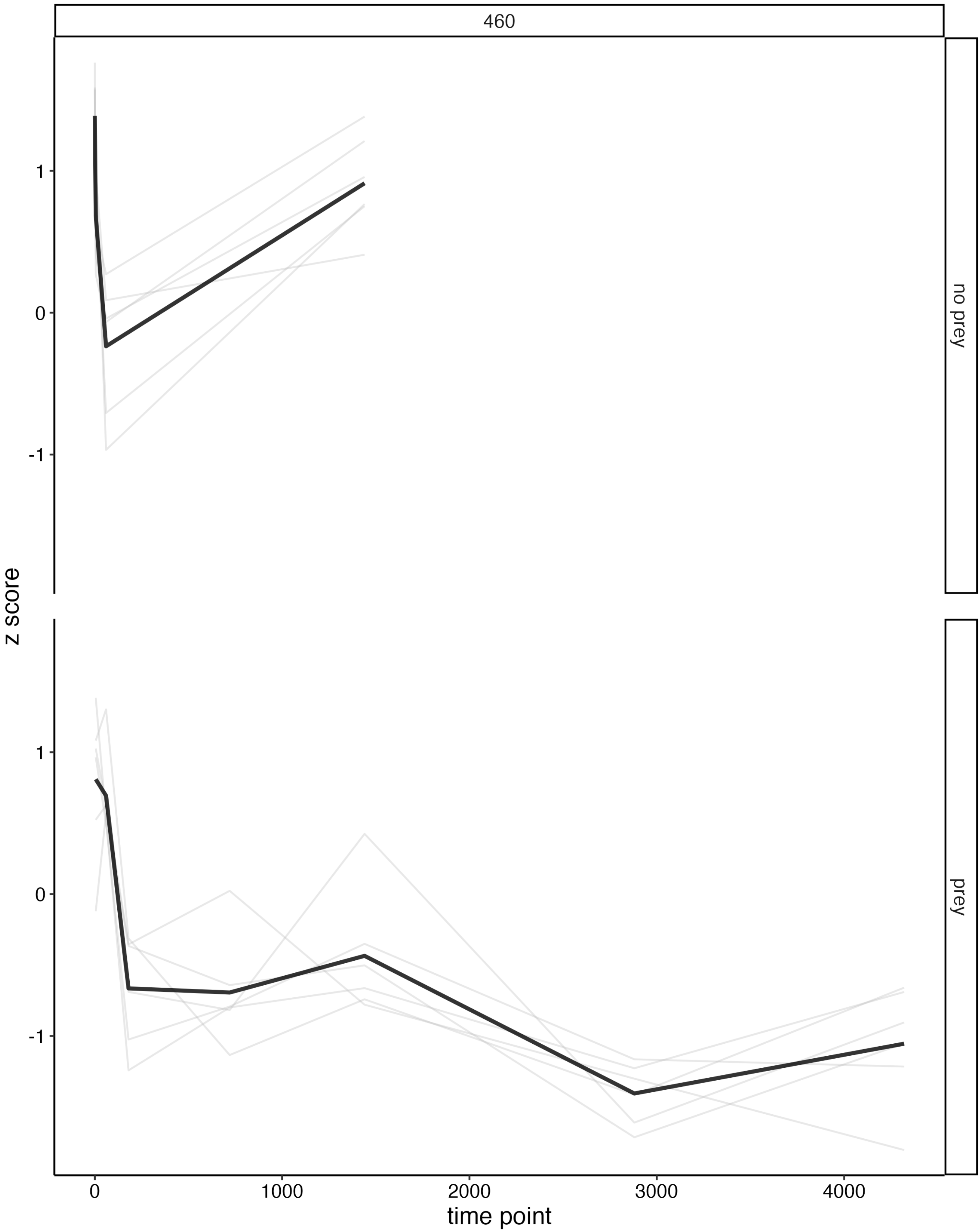

460
no prey
prey
z score
time point
1
0
-1
0
1000
2000
3000
4000

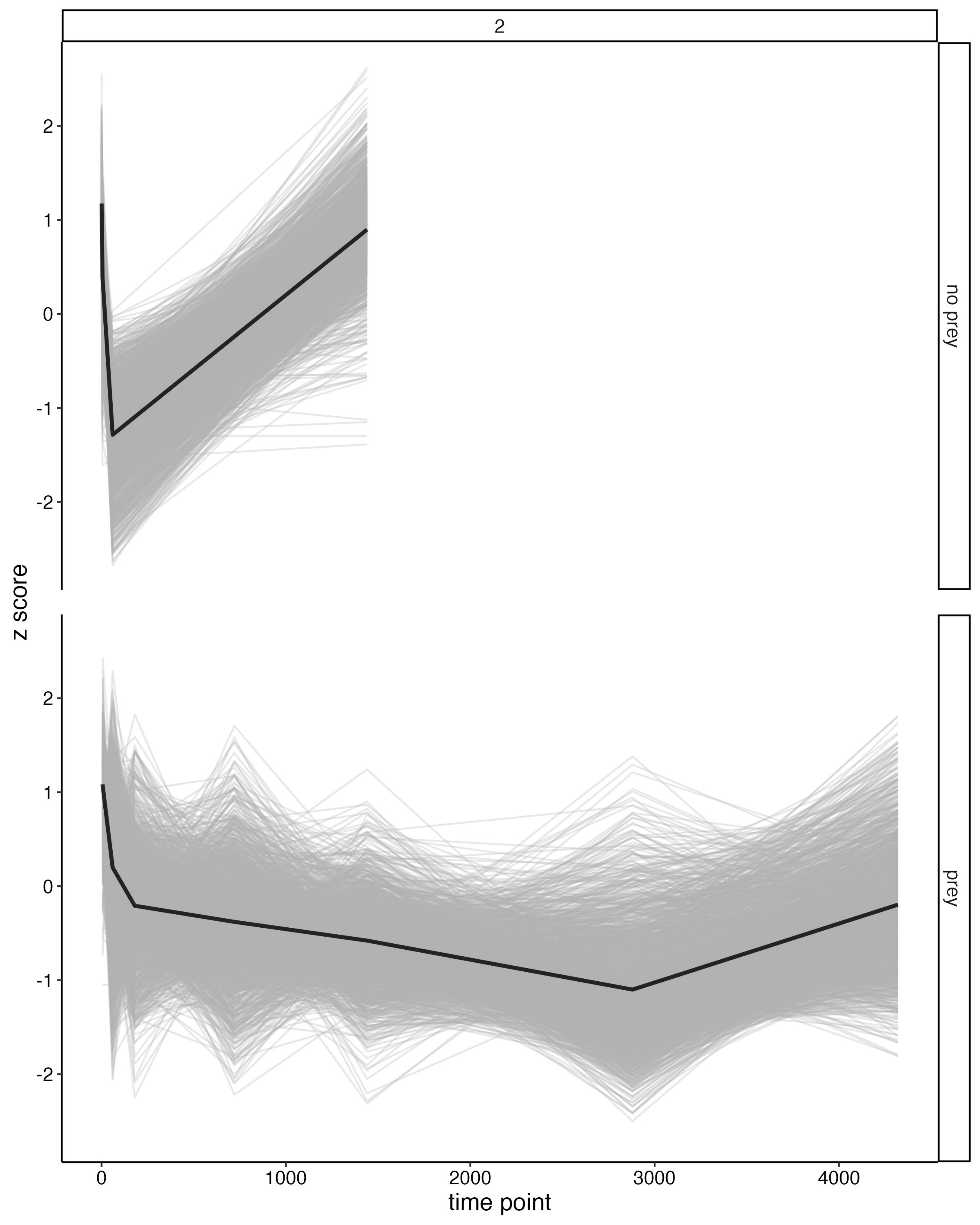

2
no prey
prey
z score
time point
2
1
0
-1
-2
0
1000
2000
3000
4000

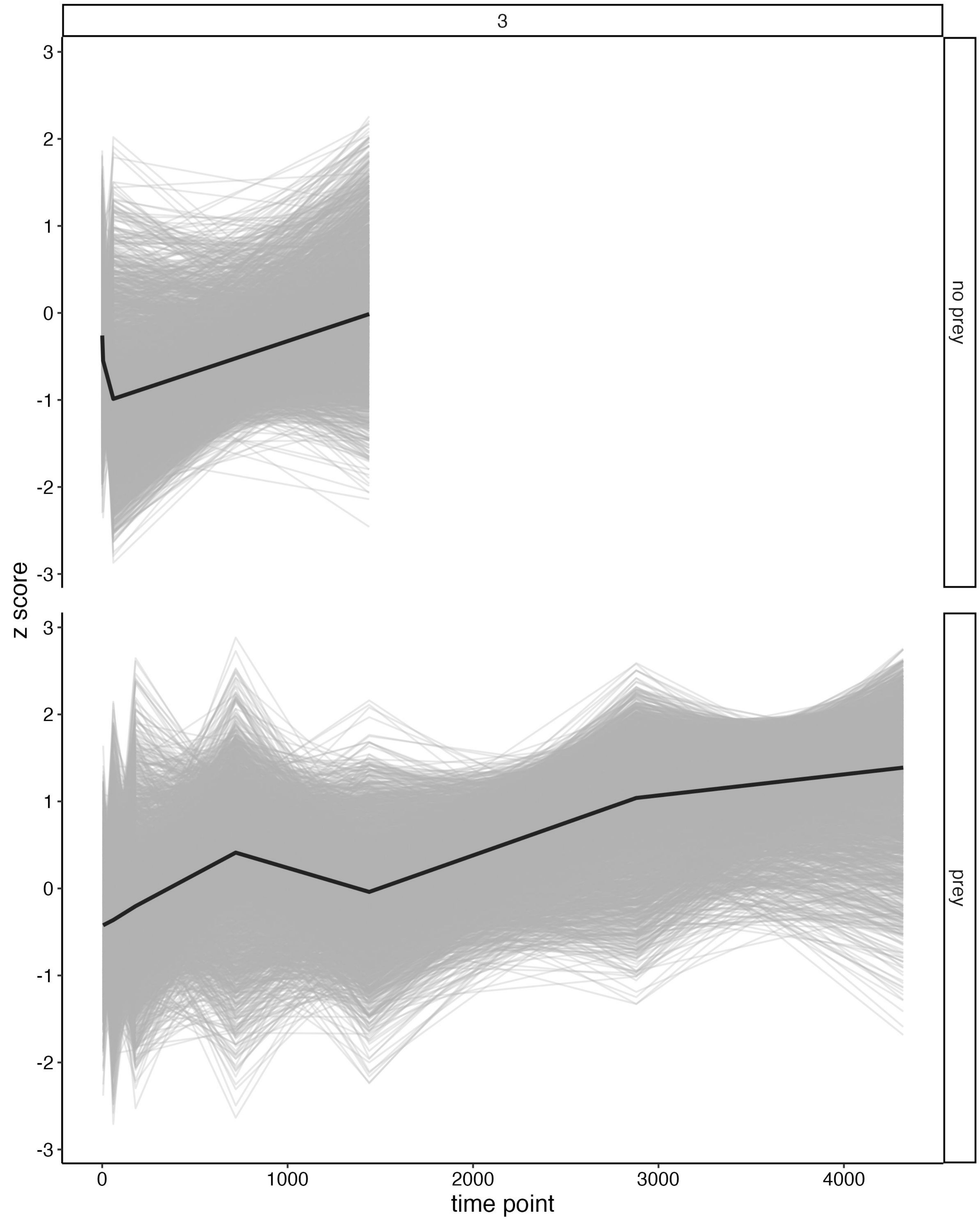

3
no prey
prey
z score
time point
3
2
1
0
-1
-2
-3
0
1000
2000
3000
4000

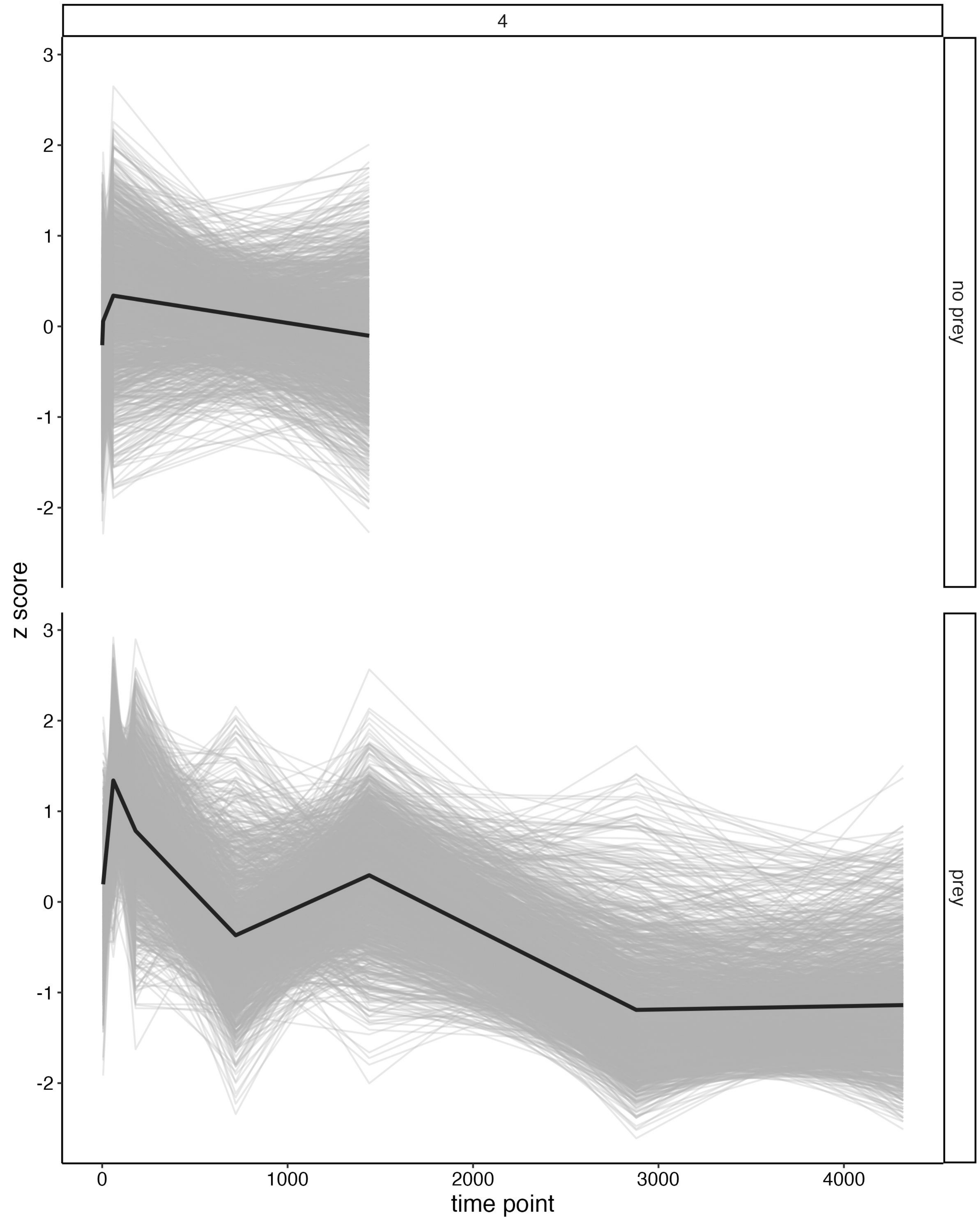
4
no prey
prey
z score
time point
3
2
1
0
-1
-2
0
1000
2000
3000
4000

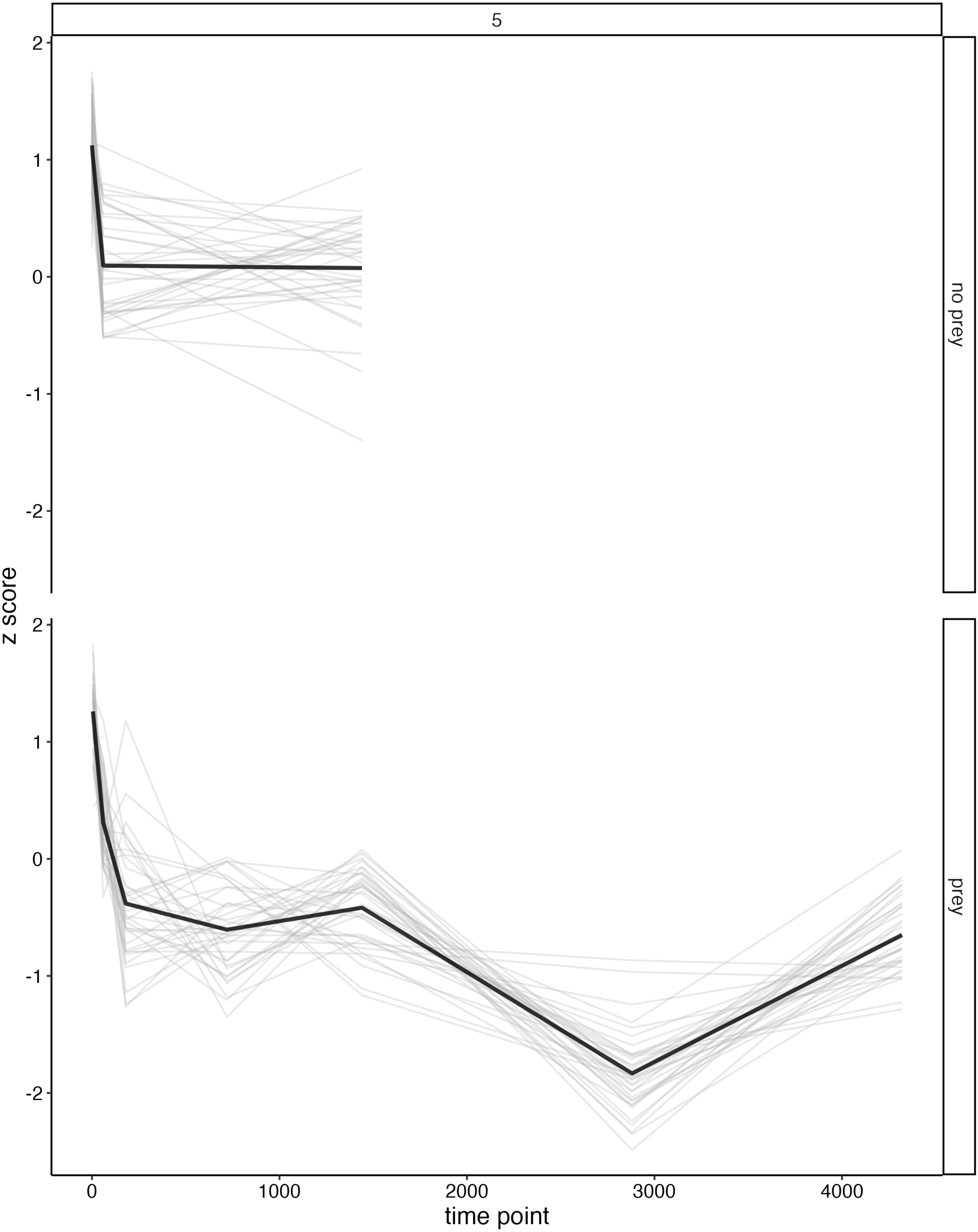

5
no prey
prey
z score
time point
2
1
0
-1
-2
0
1000
2000
3000
4000

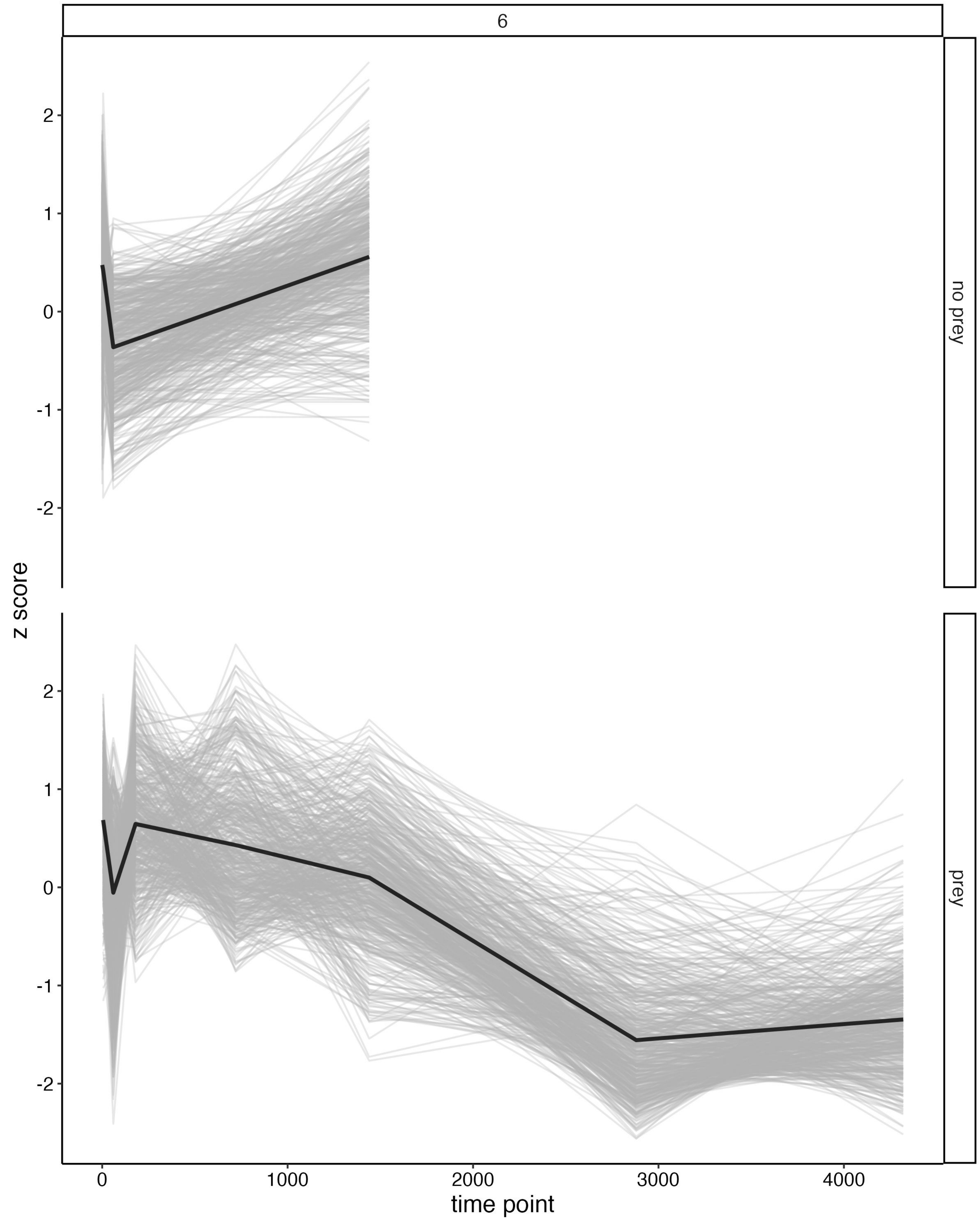
6
no prey
prey
z score
time point
2
1
0
-1
-2
0
1000
2000
3000
4000

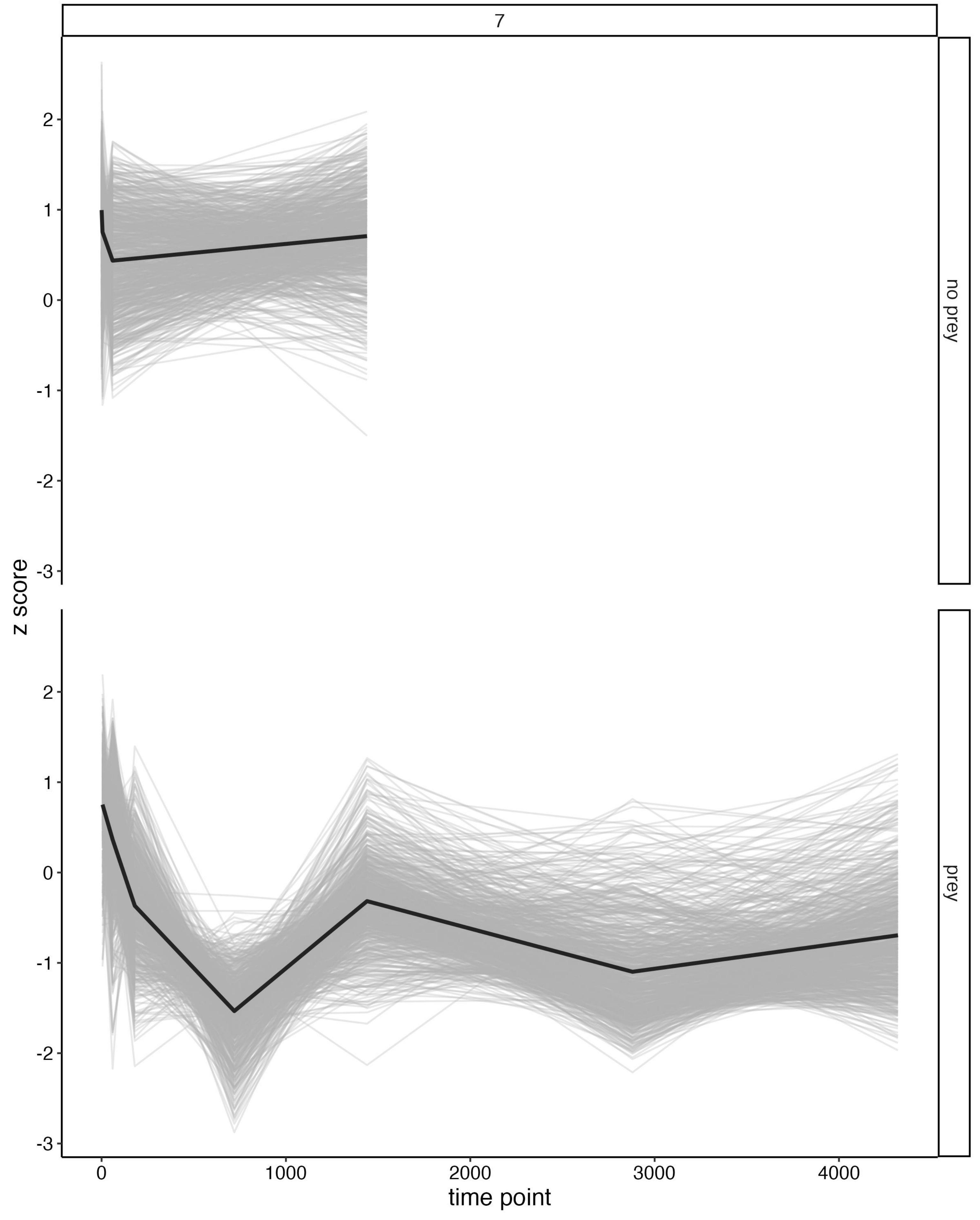

7
no prey
prey
z score
time point
2
1
0
-1
-2
-3
0
1000
2000
3000
4000

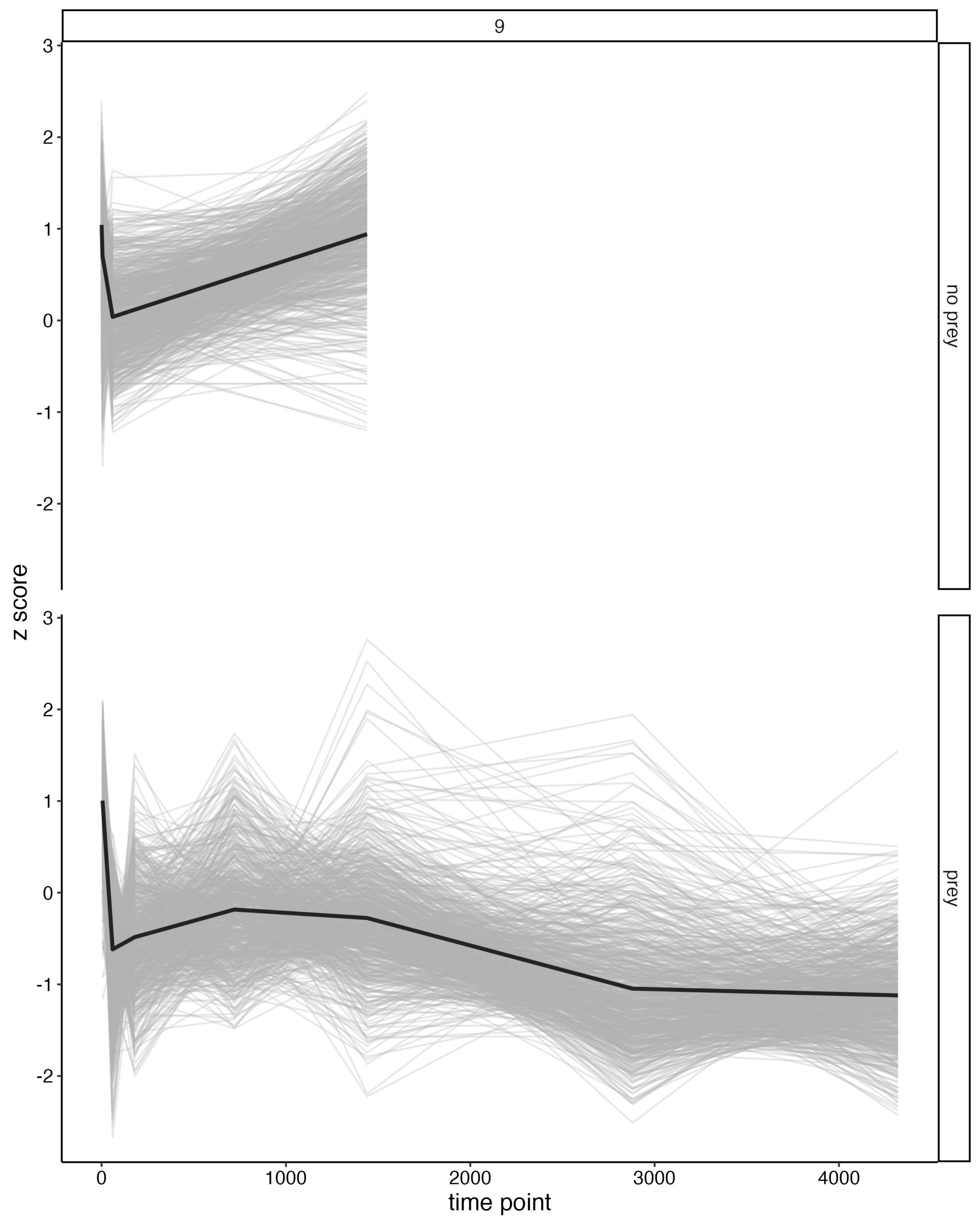

9
no prey
prey
z score
time point
3
2
1
0
-1
-2
0
1000
2000
3000
4000

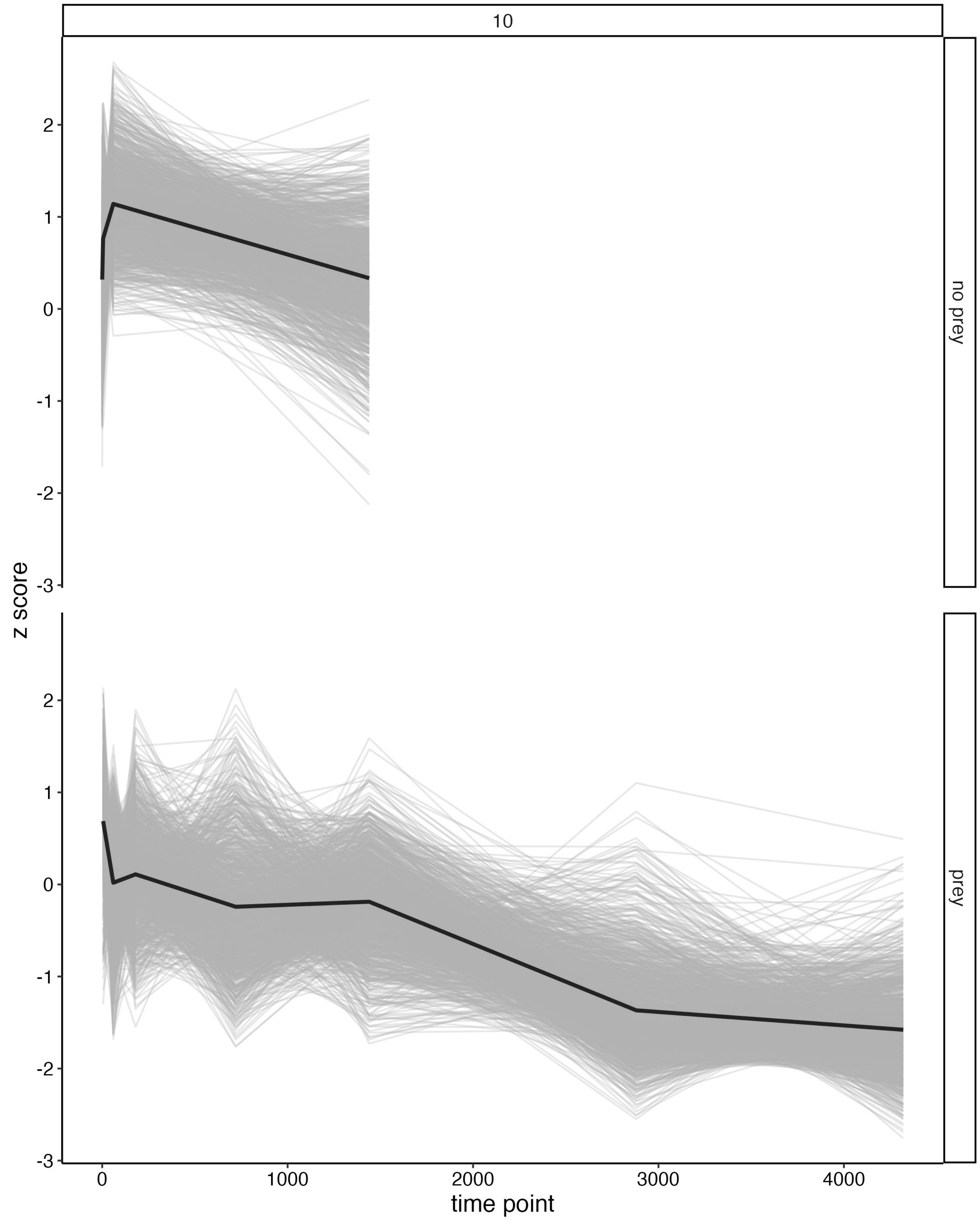
10
no prey
prey
z score
time point
2
1
0
-1
-2
-3
0
1000
2000
3000
4000

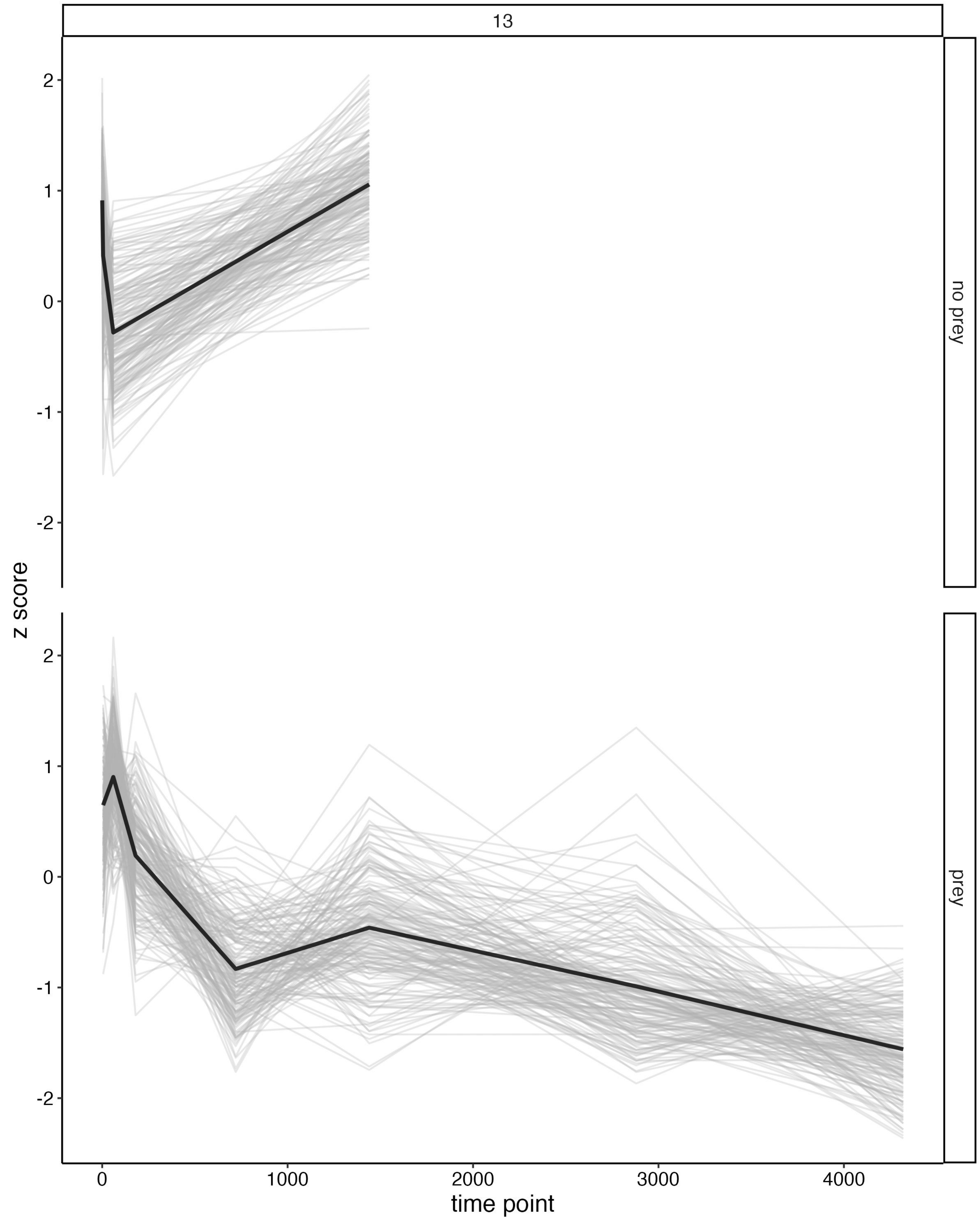

13
no prey
prey
z score
time point
2
1
0
-1
-2
0
1000
2000
3000
4000

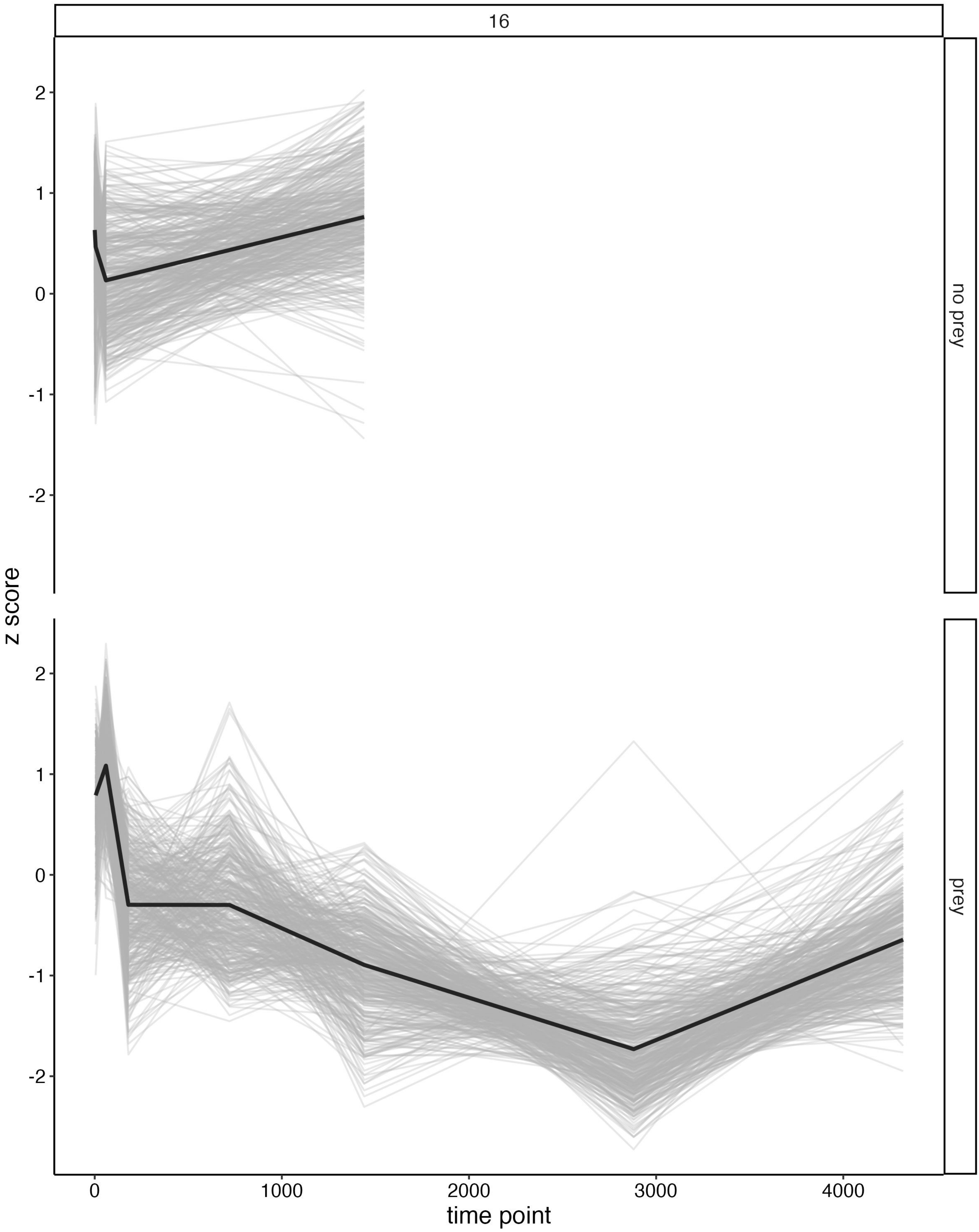
16
no prey
prey
z score
time point
2
1
0
-1
-2
0
1000
2000
3000
4000

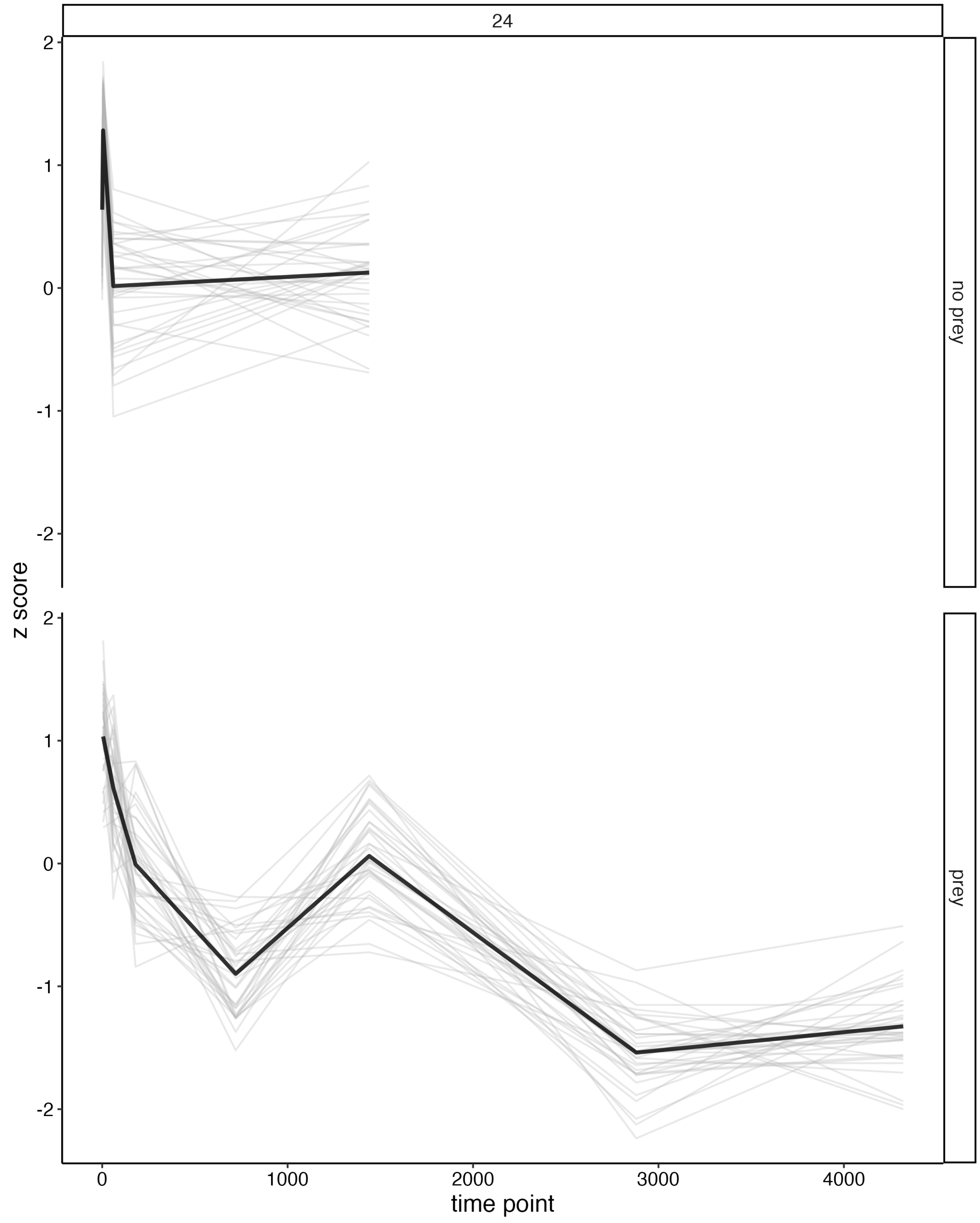
24
no prey
prey
z score
time point
2
1
0
-1
-2
0
1000
2000
3000
4000

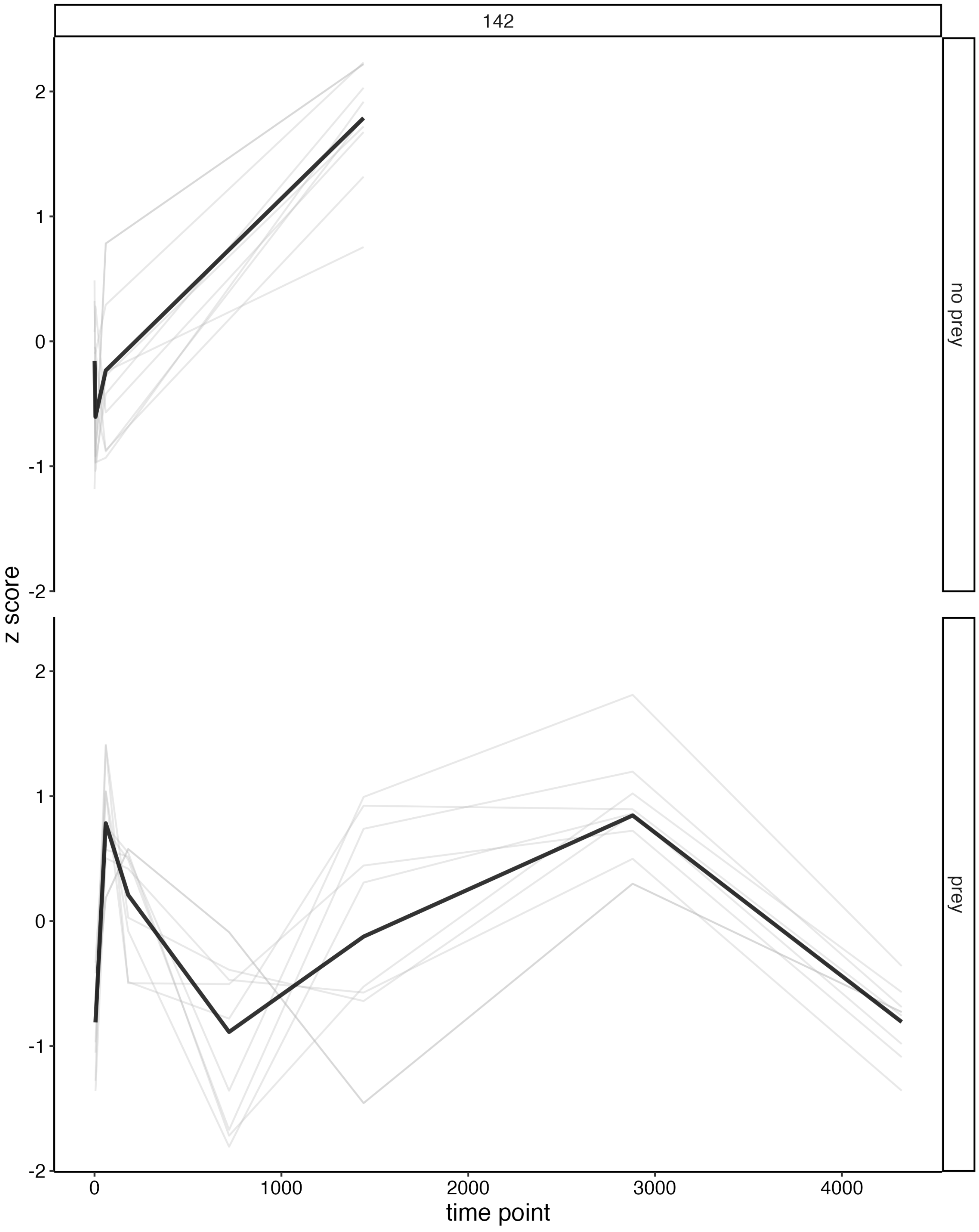

142
no prey
prey
z score
time point
2
1
0
-1
-2
0
1000
2000
3000
4000

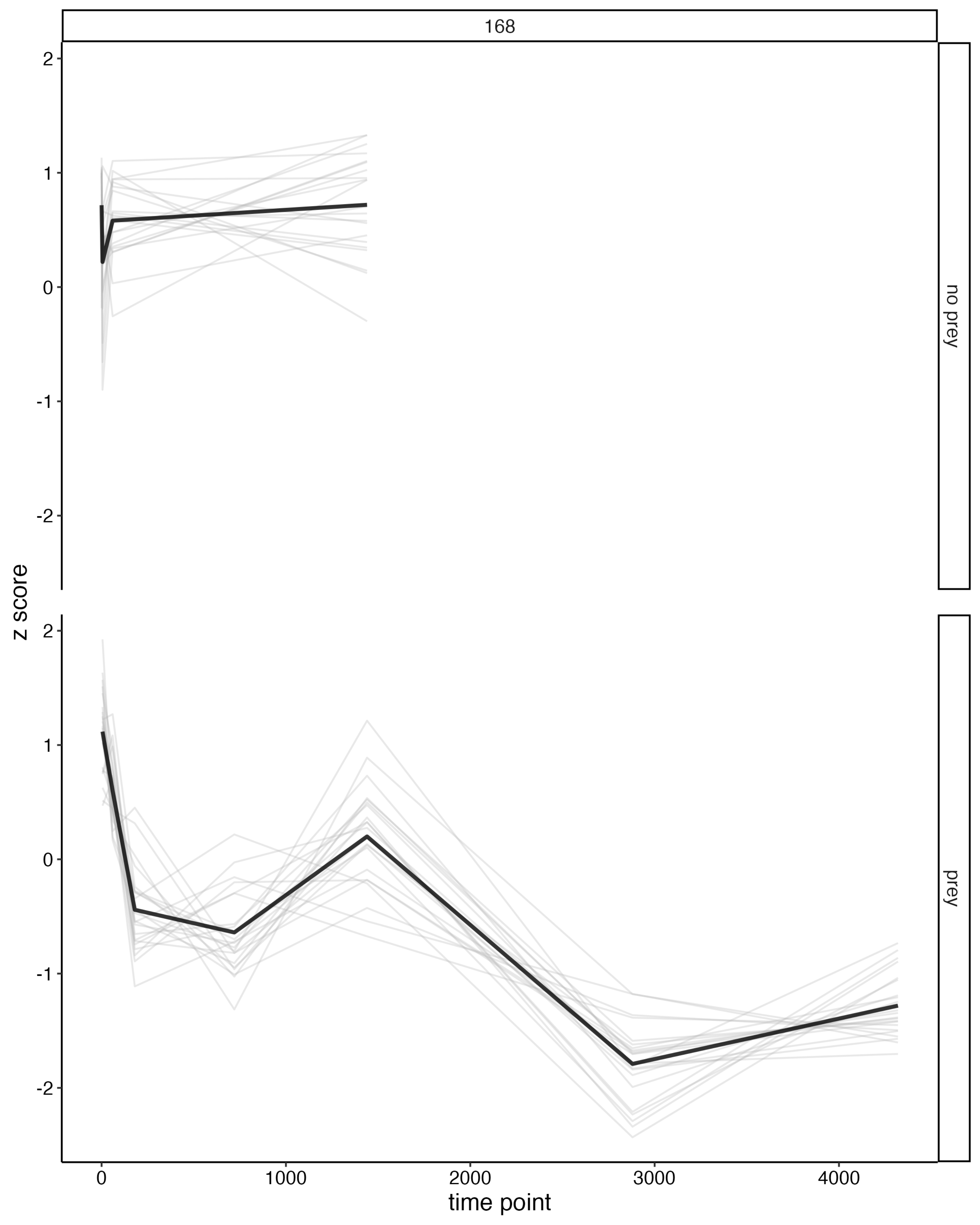

168
no prey
prey
z score
time point
2
1
0
-1
-2
0
1000
2000
3000
4000
