# Supplementary material for: Comparative transcriptomics of Venus flytrap (Dionaea muscipula) across stages of prey capture and digestion
Source: PLoS One. 2024 Aug 12;19(8):e0305117. doi: 10.1371/journal.pone.0305117 (PMC11318880; doi:10.1371/journal.pone.0305117)
Supplement: S3 Fig — Number of differentially expressed genes in each co-expression module, including (1) differentially expressed genes between traps triggered with and without prey at 1 hr and 24 hr time points. Color shows which treatment had statistically higher expression. (2) The number of differentially expressed genes at the 1 hour time point found amongst the top 8 Gene Co-Expression modules. Color shows which treatment had statistically higher expression. (3) The number of differentially expressed genes at the 24 hour time point found amongst the top 8 Gene Co-Expression modules. Color shows which treatment had statistically higher expression. (PDF) [file pone.0305117.s003.pdf]

Supplemental Information 3. Number of differentially expressed genes in each co-expression module, including (1) differentially expressed genes between traps triggered with and without prey at 1 hr and 24 hr time points. Color shows which treatment had statistically higher expression. (2) The number of differentially expressed genes at the 1 hour time point found amongst the top 8 Gene Co-Expression modules. Color shows which treatment had statistically higher expression. (3) The number of differentially expressed genes at the 24 hour time point found amongst the top 8 Gene Co-Expression modules. Color shows which treatment had statistically higher expression.

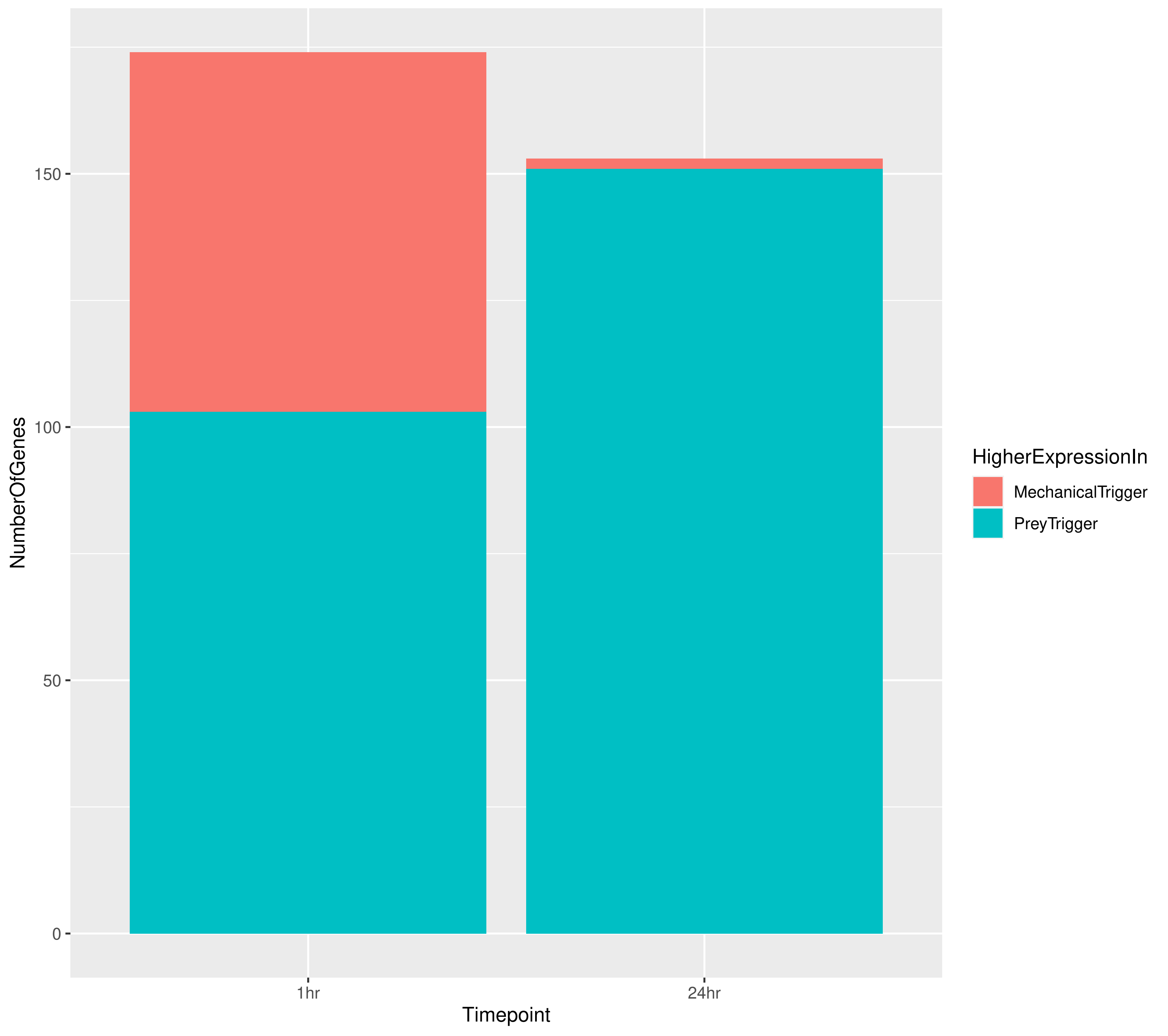
150
100
50
0
NumberOfGenes
1hr
24hr
Timepoint
HigherExpressionIn
MechanicalTrigger
PreyTrigger

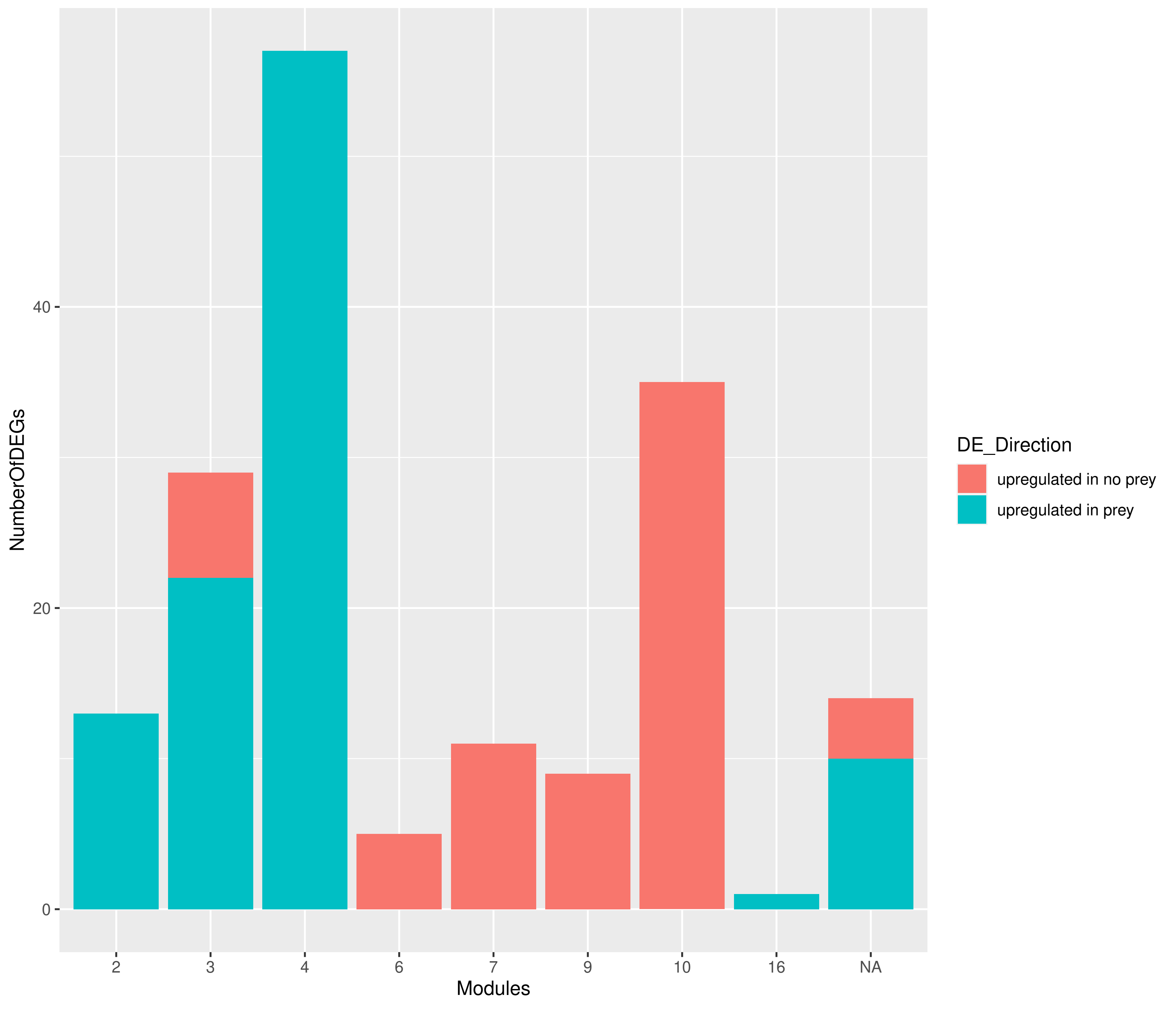
NumberOfDEGs
40
20
0
2
3
4
6
7
9
10
16
NA
Modules
DE_Direction
upregulated in no prey
upregulated in prey

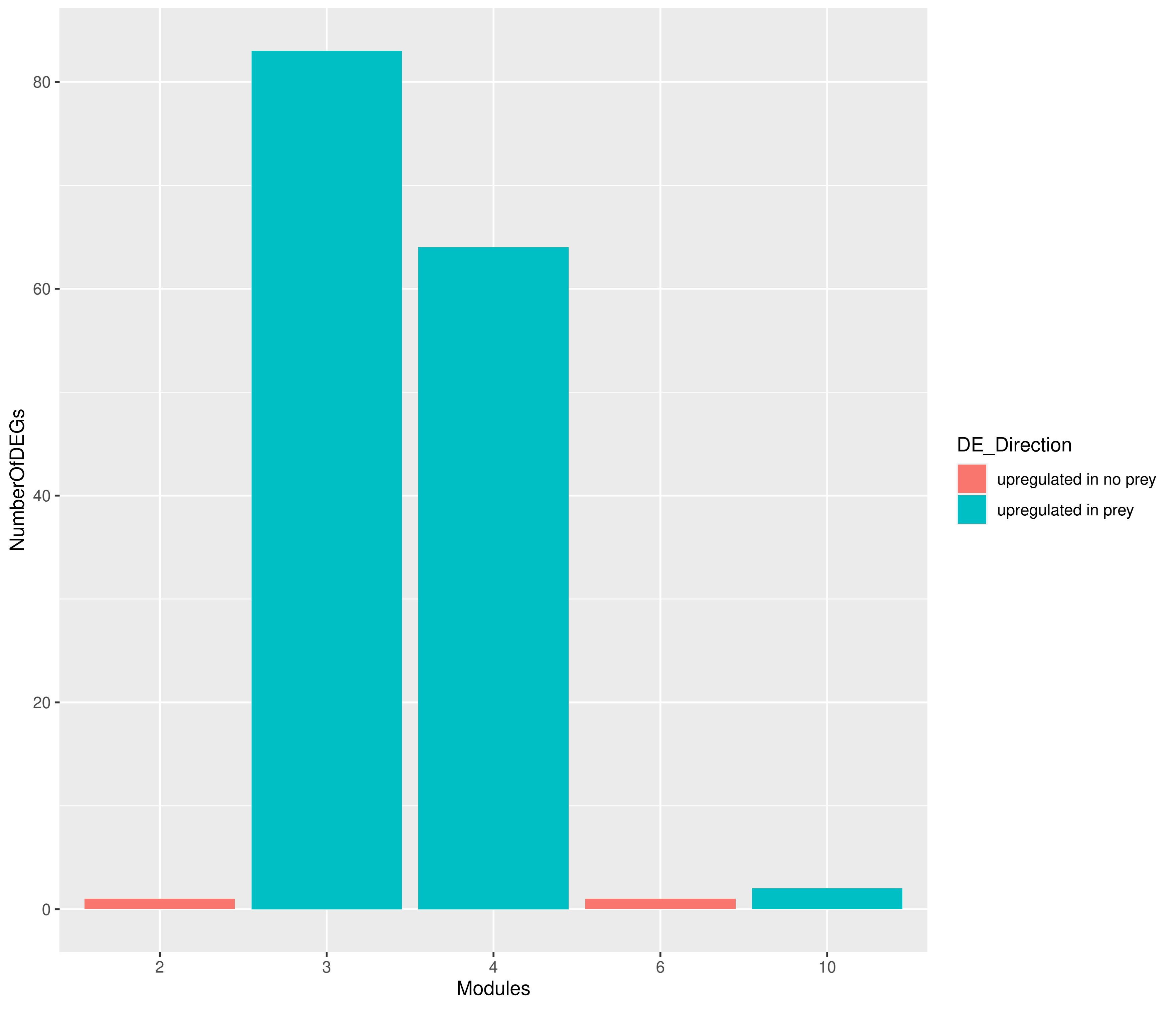

80
60
40
20
0
NumberOfDEGs
2
3
4
6
10
Modules
DE_Direction
upregulated in no prey
upregulated in prey
