# Supplementary material for: Comparative transcriptomics of Venus flytrap (Dionaea muscipula) across stages of prey capture and digestion
Source: PLoS One. 2024 Aug 12;19(8):e0305117. doi: 10.1371/journal.pone.0305117 (PMC11318880; doi:10.1371/journal.pone.0305117)
Supplement: Attachment — Submitted filename: PLOS ONE Response Letter.docx [file pone.0305117.s005.docx]

Journal requirements:

**4/22/24: Funding information removed from document.**

1. When submitting your revision, we need you to address these additional requirements.

Please ensure that your manuscript meets PLOS ONE's style requirements, including those for file naming. The PLOS ONE style templates can be found at

https://journals.plos.org/plosone/s/file?id=wjVg/PLOSOne_formatting_sample_main_body.pdf and

https://journals.plos.org/plosone/s/file?id=ba62/PLOSOne_formatting_sample_title_authors_affiliations.pdf.

**Response: Okay great.**

2. We note that the grant information you provided in the ‘Funding Information’ and ‘Financial Disclosure’ sections do not match.

When you resubmit, please ensure that you provide the correct grant numbers for the awards you received for your study in the ‘Funding Information’ section.

**Response: This has been addressed and updated in the document.**

*3. Thank you for stating the following financial disclosure:*

*[This work was supported by the Francis Marion University professional development program (119145-100000-42105-82000), The University of Georgia, and the National Science Foundation (DEB-2110875).].*

*Please state what role the funders took in the study. If the funders had no role, please state: ""The funders had no role in study design, data collection and analysis, decision to publish, or preparation of the manuscript.""*

*If this statement is not correct you must amend it as needed.*

*Please include this amended Role of Funder statement in your cover letter; we will change the online submission form on your behalf.*

**Response: This has been addressed and updated in the document.**

4. We note that Figure 1 in your submission contain copyrighted images. All PLOS content is published under the Creative Commons Attribution License (CC BY 4.0), which means that the manuscript, images, and Supporting Information files will be freely available online, and any third party is permitted to access, download, copy, distribute, and use these materials in any way, even commercially, with proper attribution. For more information, see our copyright guidelines: http://journals.plos.org/plosone/s/licenses-and-copyright.

We require you to either (1) present written permission from the copyright holder to publish these figures specifically under the CC BY 4.0 license, or (2) remove the figures from your submission:

a. You may seek permission from the original copyright holder of Figure 1 to publish the content specifically under the CC BY 4.0 license.

We recommend that you contact the original copyright holder with the Content Permission Form (http://journals.plos.org/plosone/s/file?id=7c09/content-permission-form.pdf) and the following text:

“I request permission for the open-access journal PLOS ONE to publish XXX under the Creative Commons Attribution License (CCAL) CC BY 4.0 (http://creativecommons.org/licenses/by/4.0/). Please be aware that this license allows unrestricted use and distribution, even commercially, by third parties. Please reply and provide explicit written permission to publish XXX under a CC BY license and complete the attached form.”

Please upload the completed Content Permission Form or other proof of granted permissions as an ""Other"" file with your submission.

In the figure caption of the copyrighted figure, please include the following text: “Reprinted from [ref] under a CC BY license, with permission from [name of publisher], original copyright [original copyright year].”

b. If you are unable to obtain permission from the original copyright holder to publish these figures under the CC BY 4.0 license or if the copyright holder’s requirements are incompatible with the CC BY 4.0 license, please either i) remove the figure or ii) supply a replacement figure that complies with the CC BY 4.0 license. Please check copyright information on all replacement figures and update the figure caption with source information. If applicable, please specify in the figure caption text when a figure is similar but not identical to the original image and is therefore for illustrative purposes only.

**Response: We believe you are referring to the pictures of the flytraps themselves. Those are photographs taken by first author, Jeremy D. Rentsch, who gives permission for their use. If this isn’t specifically what is meant please clarify and be specific.**

5. Please include captions for your Supporting Information files at the end of your manuscript, and update any in-text citations to match accordingly. Please see our Supporting Information guidelines for more information: http://journals.plos.org/plosone/s/supporting-information.

2. Has the statistical analysis been performed appropriately and rigorously?

Reviewer #1: Yes

Reviewer #2: No

**Response: We believe the updates to the methodology should satisfy this requirement for reviewer 2.**

4. Is the manuscript presented in an intelligible fashion and written in standard English?

PLOS ONE does not copyedit accepted manuscripts, so the language in submitted articles must be clear, correct, and unambiguous. Any typographical or grammatical errors should be corrected at revision, so please note any specific errors here.

Reviewer #1: Yes

Reviewer #2: No

**Response: No specifics provided and we found no such issues to correct in the manuscript.**

5. Review Comments to the Author

Please use the space provided to explain your answers to the questions above. You may also include additional comments for the author, including concerns about dual publication, research ethics, or publication ethics. (Please upload your review as an attachment if it exceeds 20,000 characters)

Reviewer #1: The manuscript by Rentsch et al. reports on the transcriptional responses of Venus flytrap (Dionaea muscipula) to either mechanical stimulation or by prey ingestion at various time points. By comparing the transcriptome differences at different times, the authors identified potential prey digestion- and nutrient absorption-related genes. Employing co-expression analysis, the authors clustered genes into different modules.

*1. MM should be written in more detail. How was mechanical stimulation was done? I could not find any description of it in MM or Results. How many larvae were given to each trap? How many biological replicates were harvested for each sample group?*

**Response: We have added additional details to the methodology that should remedy each of these questions.**

2. I would suggest downsizing the Discussion section. May part of Discussion which seems to be over-discussed. For example, L262-268, while AtNPR1 is indeed a crucial regulator of SA signaling, its presence does not necessarily indicate the involvement of the entire JA and SA pathway. Other evidence is needed to support that “Perhaps there is a function here in the moderation between the defense response and the wounding response”.

**Response: The discussion has been downsized significantly and the comment you reference here has been removed for being superfluous.**

3. Generally specific data, including genes and pathways, should not appear only in Discussion, e,g., AtNPR1 and AKINBETA1. These should be given in the Results (, before being discussed.

4. L242-245. Although no DEGs were detected, certain rapid responses, such as calcium signaling or MAPK signaling, may have bee activated. The trap might have perceived the triggering stimulations already, just not on the transcriptional level.

**Response: We’ve added clarity to this remark – making clear we are only speaking at the transcriptomic level and then reinforcing the idea by discussing some signaling that occurs very early.**

5. L261, please elaborate “suggest a dual role in both pathogen defense and prey digestion”. Why do PR proteins have functions in prey digestion?

**Response: Clarity has been brought to this portion. Many enzymes related to digestion are in PR gene families, but I failed to make a meaningful connection here and made a more direct connection instead.**

6. Caryophyllales already possesses ample genomic data. The authors could analyze the evolutionary history of identified candidate genes, exploring carnivory in Caryophyllales from an evolutionary perspective.

7. Please use “h” for “hour” and use “min”, but not “m” (m = meter, not minute).

**Response**: **This has been fixed throughout. Thank you.**

Reviewer #2: In this manuscript, authors compared transcriptional differences between venus flytrap with vs without prey in time series. The major achievement is that venus flytrap recognizes a successful trap after 5 min, probably starts at 1 hour, and the catabolism and nutrient transport will continue to beyond 72 hours. Briefly, authors preliminarily described the transcriptomic at three time points, without deep analysis and conclusive discovery.

**Response: Thank you for your time as a reviewer. We attempted to clarify and tighten up the manuscript to highlight a few of our exciting finds. We are excited to see what work comes of this.**

Major comments:

1, The description to methods is rough. How traps without prey are treated? “mechanical triggering” (L122) is not clear enough to understand whether this is a perfect control for traps with prey. Is there mechanical damage or only touch?

**Response: We have added some additional clarity to the methodology. This includes a more specific description of trap stimulation with a metal probe for the ‘non-prey’ treatment.**

2, for transcriptomic analysis, DEG analysis must be adjusted. In time series analysis, about 50% protein-coding genes are differently expressed. How this DEG were adjusted. There is no statistic analysis part in Methods.

**Response: Sleuth uses the Benjamini-Hochberg approach (Benjamini & Hochberg, 1995). We added a sentence and citation of B&H to the methods for clarity.**

3, L19-194 said that “the first principal component appears to correlate with the time”. No clear results support this interpretation.

**Response: We clarified the language in around this topic.**

4. The first paragraph of 3.2 (L191-197) cited Figure 2 rather than Figure 1. However, there is only one panel in Figure 2, rather than Figure 1a and Figure 1b. Please carefully check Figure 2.

**Response: Thank you for your attention on this. We fixed our error.**

5, Does the “highly coexpressed genes” (L200) mean that gene abundance is greater than five transcripts per million? Please clarify.

**Response: We clarified the language in this matter to be less ambiguous (e.g. (** **Transcript abundance profiles for these genes clustered into 14 coexpression modules, each with more than five genes)**

The font size of axis annotations in Figure 3a and 3b is too small to read.

**Response: We increased the font size and believe the figure is much improved for it.**
